# Supplementary material for: Targeting Echinococcus multilocularis PIM kinase for improving anti-parasitic chemotherapy
Source: PLoS Negl Trop Dis. 2022 Oct 3;16(10):e0010483. doi: 10.1371/journal.pntd.0010483 (PMC9560627; doi:10.1371/journal.pntd.0010483)
Supplement: S1 Fig — EmPim. (A) Amino acid sequence alignment of the kinase domains of E. multilocularis Pim (EmPim), S. mansoni Pim (SmPim), and the three human Pim isoforms (HsPim1-3). Residues identical to human Pim-1 are shown in black on grey. Kinase DFG motifs and the hinge regions are marked in red. Black triangles indicate residues known to be involved in the interaction between human Pim-1 and compound CX-6258 (numbered according to human Pim-1). (B) Phylogenetic tree based on the kinase domains of EmPim, SmPim, all three human Pim kinases (HsPIM1-3), C. elegans PRK2, and yeast PSK2. (C) Domain composition and length of EmPim, SmPim, and human Pim kinases (HsPIM1-3). The total length of the proteins is shown to the right. The positions of the kinase domain are indicated. (PDF) [file pntd.0010483.s006.pdf]

S1 Figure

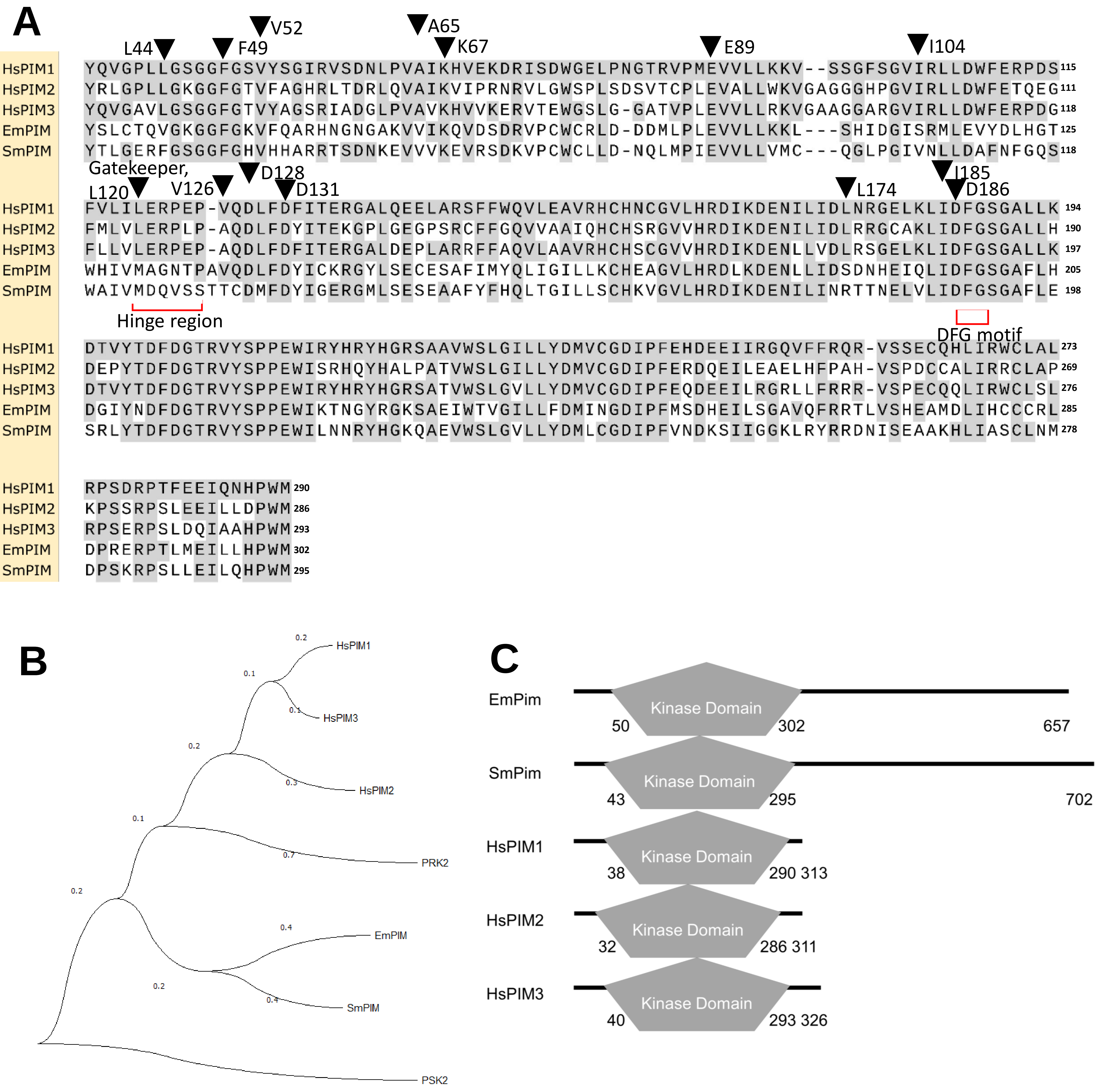

S1 Figure. Homologies and structural features of EmPim. (A) Amino acid sequence alignment of the kinase domains of *E. multilocularis* Pim (EmPim), *S. mansoni* Pim (SmPim), and the three human Pim isoforms (HsPim1-3). Residues identical to human Pim-1 are shown in black on grey. Kinase DFG motifs and the hinge regions are marked in red. Black triangles indicate residues known to be involved in the interaction between human Pim-1 and compound CX-6258 (numbered according to human Pim-1). (B) Phylogenetic tree based on the kinase domains of EmPim, SmPim, all thre human Pim kinases (HsPIM1-3), *C. elegans* PRK2, and yeast PSK2. (D) Domain composition and length of EmPim, SmPim, and human Pim kinases (HsPIM1-3). The total length of the proteins is shown to the right. The positions of the kinase domain are indicated.
