# Supplementary material for: Targeting Echinococcus multilocularis PIM kinase for improving anti-parasitic chemotherapy
Source: PLoS Negl Trop Dis. 2022 Oct 3;16(10):e0010483. doi: 10.1371/journal.pntd.0010483 (PMC9560627; doi:10.1371/journal.pntd.0010483)
Supplement: S2 Fig — Depicted are the expression values of empim according to Next Generation transcriptomic analyses performed by [29]. Values are given as transcripts per kilobase million (tpm). Shown are values for primary cells after 2 d cultivation and for MV without brood capsules (MV) as indicated. For comparison, values for the Polo like kinase encoding gene emplk1 are shown, which is strictly expressed in E. multilocularis germinative stem cells [9]. Please note that for each condition only one sample has been analyzed (n = 1). (PDF) [file pntd.0010483.s007.pdf]

S2 Figure

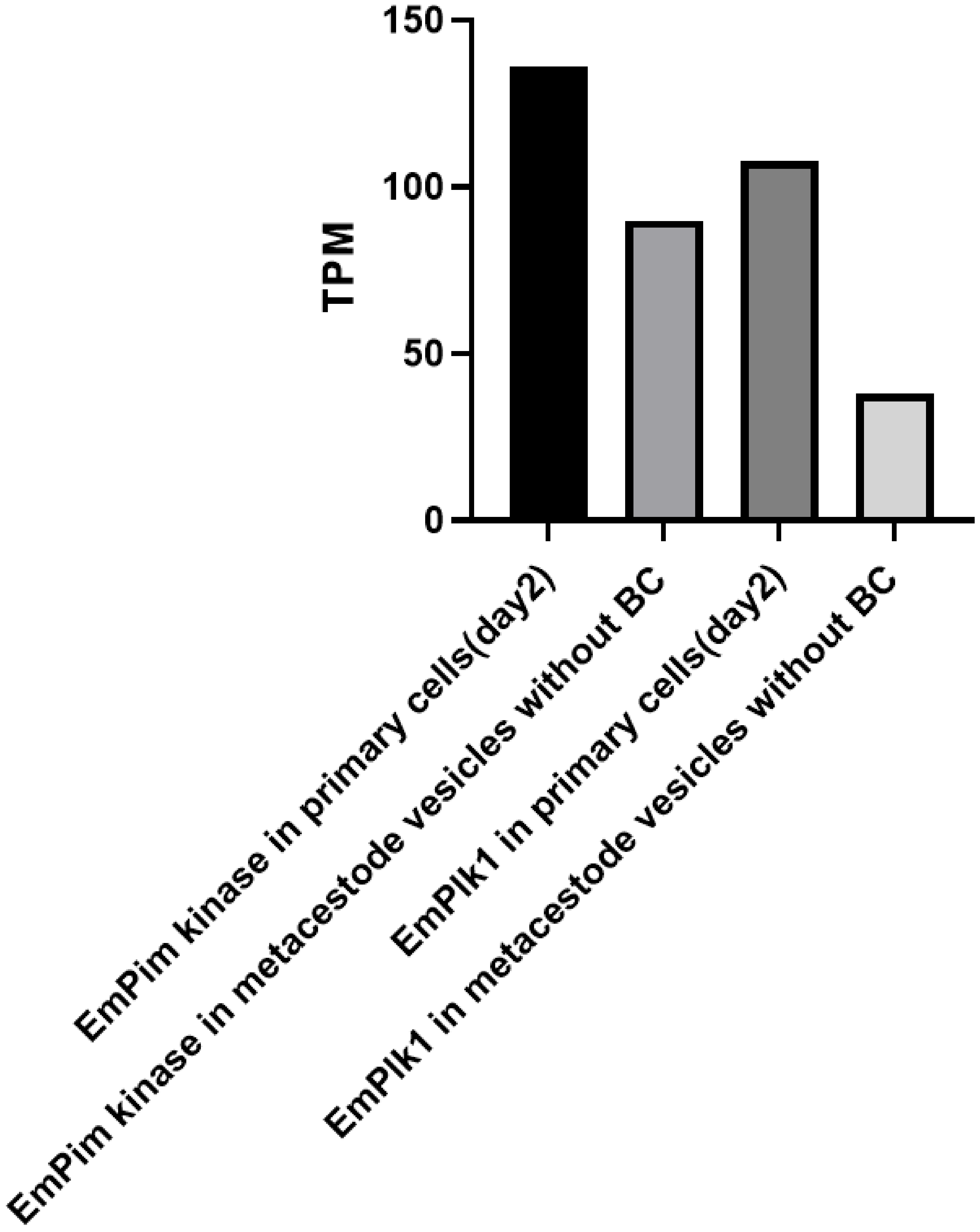

S2 Figure. Expression of *E. multilocularis empim* in larval stages. Depicted are the expression values of *empim* according to Next Generation transcriptomic analyses performed by [29]. Values are given as transcripts per kilobase million (tpm). Shown are values for primary cells after 2 d cultivation and for MV without brood capsules (MV) as indicated. For comparison, values for the Polo-like kinase encoding gene *emplk1* are shown, which is strictly expressed in *E. multilocularis* germinative stem cells [9].Please note that for each condition only one sample has been analyzed (n=1).
