# Supplementary material for: Targeting Echinococcus multilocularis PIM kinase for improving anti-parasitic chemotherapy
Source: PLoS Negl Trop Dis. 2022 Oct 3;16(10):e0010483. doi: 10.1371/journal.pntd.0010483 (PMC9560627; doi:10.1371/journal.pntd.0010483)
Supplement: S3 Fig — Image refers to microscopi images shown in Fig 3. Brown circle (distal) indicates the acellular laminated layer, thick yellow circle (proximal indicates the germinative layer of a metacestode vesicle. Blue lines indicate picture plane for stack analysis (2 μm per stack) as indicated to the right. A series of images in the germinative layer was taken as Z stack by confocal microscopy, and the image of the strongest signal (highest cell density) was analyzed. (PDF) [file pntd.0010483.s008.pdf]

S3 Figure

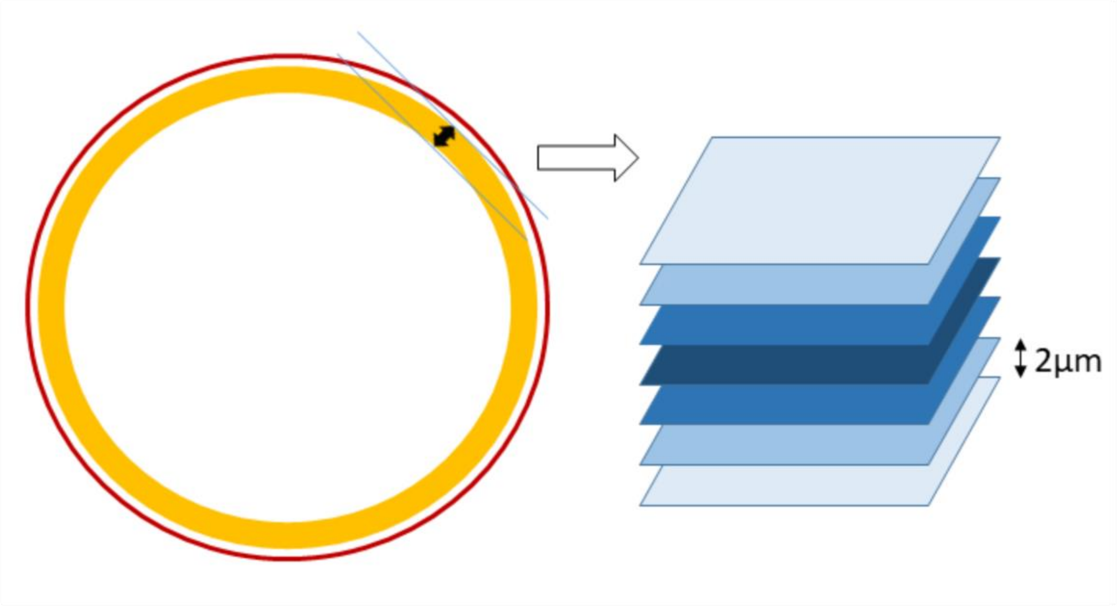

S3 Figure. Schematic image of metacestode vesicles regions analyzed in *in situ* hybridization experiments. Image refers to microscopi images shown in Fig. 3. Brown circle (distal) indicates the acellular laminated layer, thick yellow circle (proximal indicates the germinative layer of a metacestode vesicle. Blue lines indicate picture plane for stack analysis (2 µm per stack) as indicated to the right. A series of images in the germinative layer was taken as Z-stack by confocal microscopy, and the image of the strongest signal (highest cell density) was analyzed.
