# Supplementary material for: Targeting Echinococcus multilocularis PIM kinase for improving anti-parasitic chemotherapy
Source: PLoS Negl Trop Dis. 2022 Oct 3;16(10):e0010483. doi: 10.1371/journal.pntd.0010483 (PMC9560627; doi:10.1371/journal.pntd.0010483)
Supplement: S4 Fig — Cells were treated with 1 30 μM of SGI 1776 CX 6258 and Z 196138710 (as indicated) for 3 days and cell viability was measured. Signal intensities of each well were normalized to those of control samples treated with DMSO and shown as percentage. Error bar represents standard deviation. Shown are results for HEK 293 T (A) and HepG 2 (B) cells as mM concentration with LogIC50 and IC50 as indicated to the right One-Way- ANOVA test followed by Tukey’s multiple comparisons test was used for statistical analysis. (PDF) [file pntd.0010483.s009.pdf]

S4 Figure

A

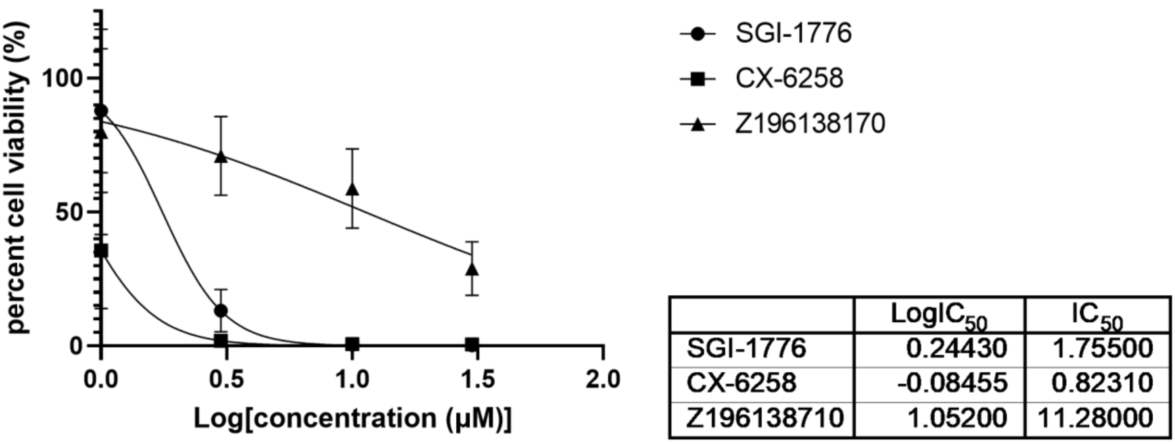

	$LogIC_{50}$	IC_{50}
SGI-1776	0.24430	1.75500
CX-6258	-0.08455	0.82310
Z196138710	1.05200	11.28000

B

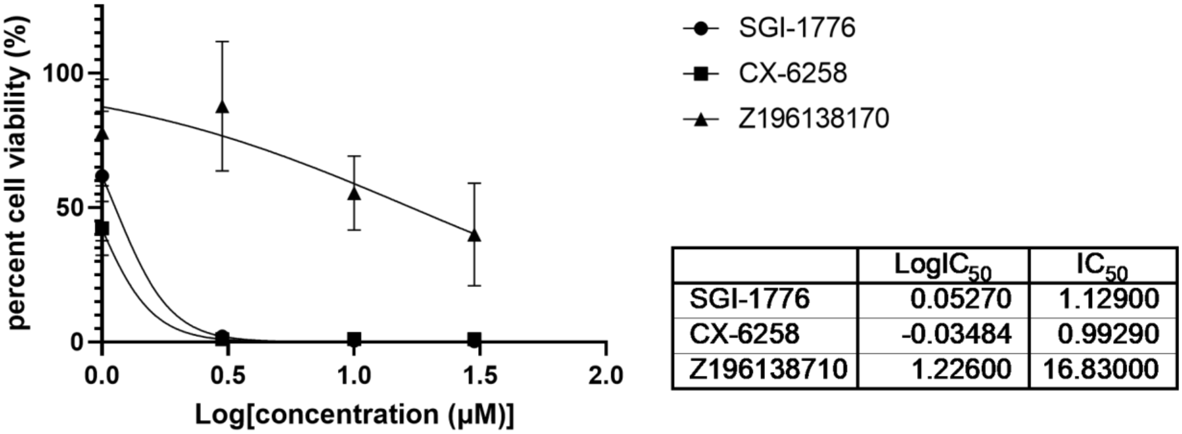

	$LogIC_{50}$	IC_{50}
SGI-1776	0.05270	1.12900
CX-6258	-0.03484	0.99290
Z196138710	1.22600	16.83000

S4 Figure: Dose response curves of Pim kinase inhibitors on human cell lines. Cells were treated with 1-30 µM of SGI-1776, CX-6258 and Z196138710 (as indicated) for 3 days and cell viability was measured. Signal intensities of each well were normalized to those of control samples treated with DMSO and shown as percentage. Error bar represents standard deviation. Shown are results for HEK293T (A) and HepG2 (B) cells as mM concentration with $LogIC_{50}$ and IC_{50} as indicated to the right. One-Way-ANOVA test followed by Tukey's multiple comparisons test was used for statistical analysis.
