# Supplementary material for: Targeting Echinococcus multilocularis PIM kinase for improving anti-parasitic chemotherapy
Source: PLoS Negl Trop Dis. 2022 Oct 3;16(10):e0010483. doi: 10.1371/journal.pntd.0010483 (PMC9560627; doi:10.1371/journal.pntd.0010483)
Supplement: S5 Fig — (A) (PDF) [file pntd.0010483.s010.pdf]

## S5 Figure

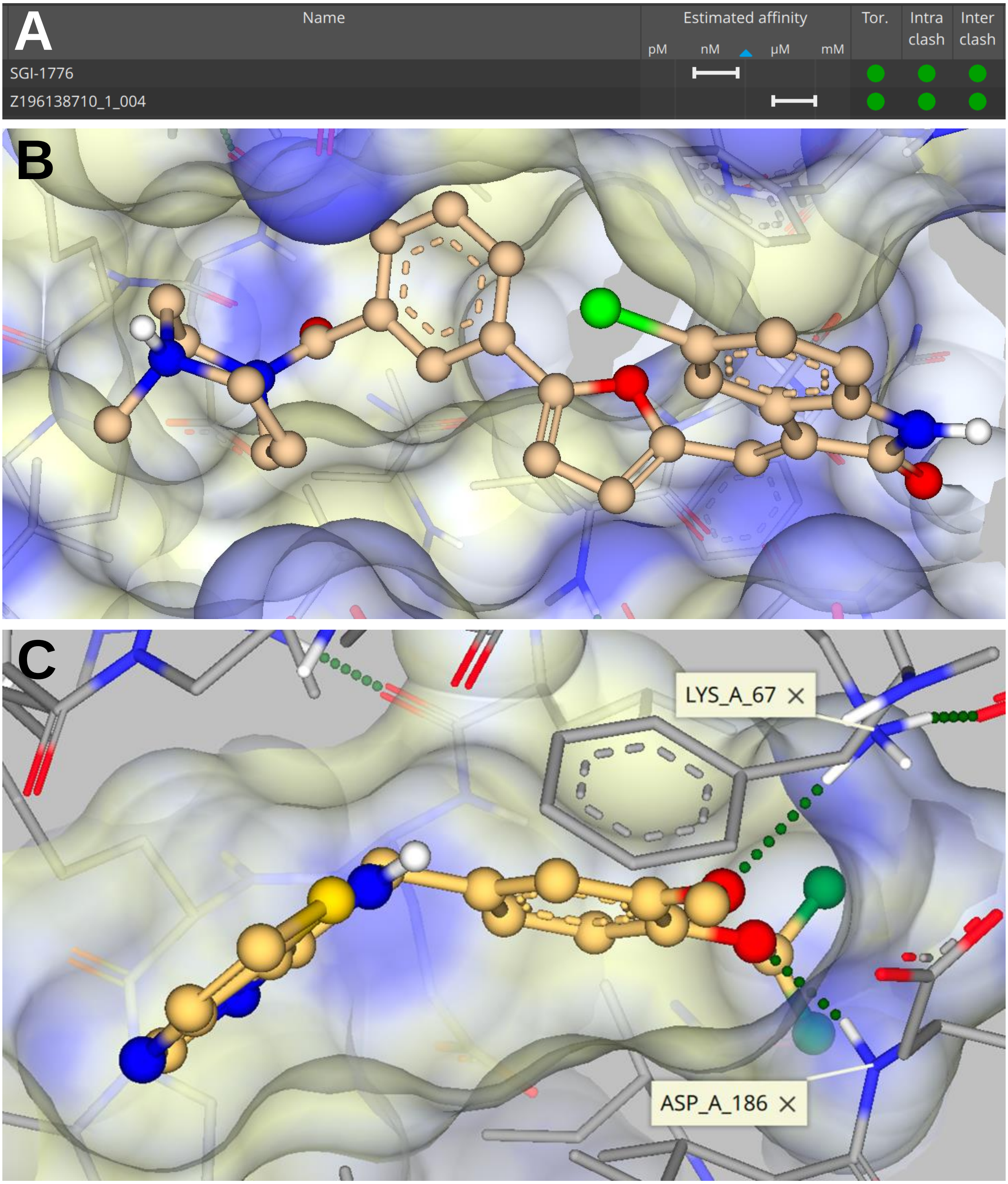


**S5 Figure. SeeSAR modelling analysis of compound binding to human Pim-1.**
(A) Output of SeeSAR analysis for estimated binding affinities of SGI-1776 and Z196138710 into the ATP binding pocket of human Pim-1. (B) Model of SGI-1776 binding into the Pim-1 ATP binding pocket. (C) Model of Z196138710 binding into the ATP binding pocket of human Pim-1.
