# Supplementary material for: Targeting Echinococcus multilocularis PIM kinase for improving anti-parasitic chemotherapy
Source: PLoS Negl Trop Dis. 2022 Oct 3;16(10):e0010483. doi: 10.1371/journal.pntd.0010483 (PMC9560627; doi:10.1371/journal.pntd.0010483)
Supplement: Attachment — Submitted filename: Response to reviews.docx [file pntd.0010483.s011.docx]

**PNTD-D-22-00613**

**Response to Reviews**

All original comments of reviewers and editors are printed in *italics*. All our comments (responses) are highlighted in blue.

***Editor’s comments:***

*The reviewers felt that the manuscript was interesting, well-written and relevant to the field. They have provided useful suggestions in terms of writing more detailed descriptions of how the vesicles were prepared, generating an IC50 curve for Z196138710, and adding any potential data on in vivo toxicity. It would be preferred if additional experiments could be performed to test the in vivo toxicity, but as the reviewers indicate, please at least search and describe PK data, where available.*

**Response:** As outlined in more detail below, we performed additional experiments and now provide an IC50 curve for Z196138710 on human cell lines and included/discussed additional available information on pharmacokinetics of PIM inhibitors. We agree that in vivo data would be informative but, as also outlined in more detail below, we already argued in the original manuscript and we do so in the revision that Z196138710 is still too experimental as to justify the sacrifice of experimental animals (apart from the fact that 30 days for the revision, even with added extra time, would not have been sufficient to carry out these experiments nor even to obtain permission for these experiments). As already outlined in the original manuscript, we never claimed that Z196138710 is a replacement for current therapeutic regimes. Our study instead focussed on novel, bioinformatic methods to identify potential targets and respective lead compounds. We discussed this in more detail in the revised version. We also think that our work contains sufficient work and original data to justify publication as an original paper, even without in vivo data.

*Please also ensure that statistical tests are briefly described in each of the in the figure captions where relevant (Fig 3B, 5B, 6A, 6C, 7B-F). Although most of these are described in the text, 3B is does not seem to be, and the rest could just be identified with, for example, “(one way ANOVA, with Tukey’s multiple comparisons test)” after the P value descriptions. Additionally, reviewer 2 recommends separating the results and discussion. While that may improve clarity somewhat, it would be acceptable to submit these combined as currently written, and that editing decision will be left up to the authors.*

**Response:** We now added additional information on statistical tests in all cases (please see below for more details). We originally had written a manuscript in which results and discussion were separate, somehow according to the suggestion of the reviewer, but already prior to submission we felt that a combined results/discussion section would be more appropriate in this case. We still stick to this opinion and, thus, kept results and discussion still combined in the revised version. We hope reviewers and editors can accept that.


***Reviewer's Responses to Questions***

**Remark:** We want to thank all reviewers for their investment of time and efforts towards improving our manuscript and work. It is nowadays very difficult to find qualified reviewers and we very much appreciate their dedication.

***Key Review Criteria Required for Acceptance?

Methods***

*Reviewer #1: all: yes, except two missing information about statistics, as indicated below.*

**Response:** Thank you for the ‘yes’. We respond to the rest in detail below.

*Reviewer #2: This is an interesting paper demonstrating the identification of a novel inhibitors of PIM kinase, which is found in stem cells of E. multilocularis metacestodes and is involved in cell cycle progression. The authors also demonstrate the interaction between PIM and CDC25. The paper is especially interesting due to the fact that it shows how inhibitors of human kinases can be further investigated through high-throughput in silico docking approaches, ending up with a novel inhibitor that has increased specificity for the parasite kinase over the human kinase.
The study has been carried out with great care and expertise. Overall the paper shows good science, as expected from this group, and is very well written.*

**Response:** We thank the reviewer for this kind assessment.

Reviewer #3: Yes, the manuscript met all the criteria mentioned for methods

**Response:** Thank you for pointing this out.
--------------------

***Results***

*Reviewer #1: all: yes.*

**Response:** Again, thank you for the ‘yes’.

*Reviewer #2: Overall this is a highly interesting contribution to the field. The results are clearly presented, and this includes the background of supplementary files. Figures and Tables are clearly described.
The major backdrop of this paper is the lack of any in vivo data, which would confirm that the hopes that are being raised by the discovery that this group has made are actually justified.
The drug discovery field is full of studies that show promising results in vitro, which are then often not translated into an animal model. More concretely for this study, we don't even know whether application of the novel compound to mice would kill these animals or not. If there is some background information available on that, the authors should show it.*

**Response:** We agree with the reviewer that the literature is full of in vitro studies concerning potential drugs and drug targets and that finding an eventually effective medication is a difficult task. While, from the technical point of view, it is of course desirable to have animal experimentation data for all potential drug candidates, we also must weigh this up in the context of animal welfare (actually, by EU and German law we are obliged to do so). We thus propose that prior to animal experimentation, promising targets should be found and that modern methodology should be applied to find compounds that have the potential to discriminate between parasite and host targets. This is what we did in our work – as a first step in the case of Pim kinases. We critically checked our manuscript for any passages that might be taken as anchors for false glimmers of hope but, honestly, we did not find them (‘hope-raising’, admittedly, is a very subjective topic). We merely stated that we ‘demonstrated high throughput in silico approaches as useful methodology in finding lead compounds’ (lines 45-46, original manuscript) and that we propose the parasite’s PIM kinase as a promising target (lines 47-48, original manuscript). We therefore strongly believe that we did not over-interpret the meaning of our data but, rather, provided a stone-cold sober analysis. We therefore kept the respective wording in our revised version.

As correctly pointed out by the reviewer, we do not yet know whether Z196138710 would kill mammals (possibly by off-target effects). This is the reason why local authorities who are in charge of animal experimentation approvals ask for a maximum of preparatory in vitro data to minimize the risk of unnecessary animal use. We therefore proposed in our original manuscript that additional experiments (e.g. kinase activity assays) must be performed to further assess the potential of Z196138710 of being active against the parasite and non-toxic to mammals. We do not think that the local authorities are over-eager in following the EU guidelines for animal welfare and, thus, refrained from doing in vivo experiments at the present time point. Once the respective data are available, it will be possible to obtain animal experimentation clearance (which, by the way, would take longer than the experiments themselves – at least where we are situated).

*Reviewer #3: Yes, the manuscript met all the criteria mentioned for results. Minor changes are suggested for Figures*

**Response:** We will address this point in more detail below.

--------------------

***Conclusions***

*Reviewer #1: all: yes.*
**Response:** Thank you.

*Reviewer #2: The major backdrop of this paper is the lack of any in vivo data, which would confirm that the hopes that are being raised by the discovery that this group has made are actually justified.
The drug discovery field is full of studies that show promising results in vitro, which are then often not translated into an animal model. There are a thousand reasons why a compound will not work in an animal. More concretely for this study, we don't even know whether application of the novel compound to mice would kill these animals or not. If there is some background information available on that, the authors should show it. One of these compounds has been in phase 1 studies, and I am sure there is data providing information on PK properties of this drug, just to see whether the concentrations used in this paper are actually realistic. I believe such data should be discussed.
Inclusion of a small in vivo efficacy study, even if it turn out negative (e.g. the drugs has no or only limited effect in a mouse model), would give the reader a better understanding of the importance of the message that is conveyed here, and would surely make this study much more interesting.*

**Response:** For comments on the first part of the revewier’s critique, please see above. Concerning the second part, we included and discussed available in vivo data in the revised version of the manuscript (lines 777 - 801). Concerning the last sentence of the reviewer: according to the instructions of this journal, we are obliged to carry out animal experimentation according to ‘relevant national and international guidelines’. We are aware that the interpretation of international guidelines found quite variable impact on legal wording in national guidelines. For us, the German ‘Tierschutzgesetz’ (animal protection law) is applicable and according to §7 Tierschutzgesetz, we must limit animal experimentation to *strictly essential measures*. This notion, which is upheld by the local authorities (according to §8 Tierschutzgesetz), does not embrace the concept of *small in vivo efficacy studies which might turn out negative but are nevertheless informative*. Yet again, we think that our work contains sufficient valid data to be interesting for the readers of this journal, that these data are sufficient for and original paper in this journal, and we think that sacrificing animals at the present time point is too premature.

*Reviewer #3: Yes, the manuscript met all the criteria mentioned for conclusions.*
**Response:** Thank you.
--------------------

***Editorial and Data Presentation Modifications?***  *Reviewer #1: Only minor improvements required*
**Response:** Those are addressed below.

*Reviewer #3: Line 91: I prefer the word “niche” however the word department seems to be right.*
**Response:** Actually, ‘niche’ is wrong. We changed it to ‘population’, which should be clear.

*Line 148: a dot is missing in “(GH09) [30]The”*
**Response:** corrected.

*Line 166: the abbreviature for days was already introduced in line 154. Please replace days by d*

**Response:** corrected.

*Paragraph starting at line 176: In my opinion, the authors take for granted that at higher luminescence more viability but, How do you know this?*

**Response:** The Cell Titer Glo 2.0 assay employed in our work has previously been shown to produce luminescent values that are directly proportional to the amount of ATP and the number of viable cells in culture vials. We explained this in the M&M section (lines186 - 189) and added three references in which this relation has been shown previously.

*Line 281: WormBase ParaSite, with capital letter in b.*
**Response:** corrected.

*Line 283: Domain instead of Dmain.*
**Response:** corrected.

*Line 313: replace seeSAR with SeeSAR.*

**Response:** corrected.

*Figure 1: for the sake of clarity, the pim kinase sould be remarked.*

**Response:** We did so.

*Figure 1: what do the percentages below the heatmap means? percentage of total viability? please, clarify.*

**Response:** To put it short, they mean ‘viability’. We added a more detailed explanation to the legend of Fig. 1.

 *Figure 3: I like the pictures but I wonder if some kind of small drawing could be included showing schematically in which slice/position of the MV the pictures were taken*

**Response:** We added a supplementary figure (S3 Figure) depicting the metacestode region from which the microscopic images derive.

*S1 Figure: the structure for the compound Z991902128 can´t be seen (please see file 17).*

**Response:** The reviewer is right. This point has escaped us. We corrected it.

*S2 Figure: for the sake of clarity, perhaps “expression levels of empim RNA in..” sounds clearer.*
**Response:** we corrected this.
--------------------

***Summary and General Comments***

*Reviewer #1: The paper of Koike et al. deals with a PIM kinase of E. multilocularis as potential target for chemotherapy. In this comprehensive study, the authors provide in situ hybridization data showing stem cell activity of EmPIM. Y2H analyses revealed its interaction with E. multilocularis CDC25, a cell cycle regulator. Compounds known to inhibit human PIM caused deleterious effects on metacestode vesicles in vitro by preventing further development. Furthermore, the authors performed a high-throughput in silico modelling approach and identified a compound called Z196138710. This substance also affected cultured metacestode vesicles in vitro but showed less toxicity towards human HEK293T and HepG2 cells. The authors conclude that EmPIM is a promising target, and their study led to the identification of a novel small molecule compound with high effectivity against EmPIM and, thus, E. multilocularis.
The manuscript is very well written, and its content perfectly fits to the aims and scope of PLoS NTD. As such, it will be of high interest for the community. I have only little to suggest for improvement.*

*Minor comments
line 96: define PK as abbreviation for protein kinase, and use it from this point on.*

**Response:** We made it so.

*line 615: Fluence not in italics (as elsewhere in the text; see also lines 295-307)*
**Response:** We made it so.

*Fig. 3, B: mentioned is “statistical analysis” but there is no statistic method mentioned, nor statistic values or indicators in the figure.* 
**Response:** The reviewer is right. It was incorrect to use the term ‘statistical analysis’ since we only reported the average numbers of EdU+/empim+ cells per mm2 of germinal layer without any comparison. We corrected the legend accordingly.

*Fig. 4/S1 Fig.: Is it really necessary to go with both figures? It seems there is enough overlap to fuse contents to one figure.*

**Response:** We had the same impression and, prior to submission, we fiddled a lot to bring in line the amount of information needed and the requirements of PNTD maximum figure sizes. To put it blandly, the answer is: YES, it is necessary. We hope the reviewer can accept this.

*S2 Fig.: is this an n=1 experiment? If yes, indicate. If not, any statistics available?*
**Response:** Yes, this is an n=1 experiment. We added this information to the legend.

*References: harmonize the mixture of capital and small letter writing for paper titles according to the instructions for authors (see e.g. reference 48 vs others)*
**Response:** We made it so.

*Reviewer #2: I have few points that the authors might want to consider in their revision:
Abstract and introduction

The authors indicate that it is necessary to identify novel compounds for the treatment of AE. This is of course undisputed, but fact is that benzimdazoles, after being applied for many decades, are made looking worse than they actually are. To state that they do not act parasiticidal is an oversimplification of the situation. This has been shown in a study carried out many years ago on 34 patients undergoing long-term albendazole treatment (2–25 years), which showed that after treatment stop 11 of 34 patients exhibited no recurrence of disease within 16–82 months, as determined by PT, CT scanning and serology, with immunocompetence being an important criterion for parasiticidal effects (Ammann et al, PLoS Negl. Trop. Dis. 9, e0003964), while two-thirds of AE cases were not cured. Clearly, liver damage due to extended treatment is a major concern, which renders this study an important one.*

**Response:** The reviewer made a valid objection. While we mentioned in the introduction ‘high recurrence rates’, which is surely in line with the results of Amman et al. (2015), we also stated that ‘BZ are parasitostatic only’, which is not fully reflecting the situation given the results of Amman et al., and which is clearly wrong, given that it includes all benzimidazoles (even those which have not been tested against the parasite). We thus modified the text in the introduction section accordingly and included Amman et al. (2015) in the reference list.

*More specific comments:

Lane 320 ff: the initialscreen was done at one concentration only. Normally screenings are performed with different concentrations to study dose-responses, and then it would be possible to get an EC50 and a MIC, which would be much more informative. Maybe this is methodologically not possible in this system? The authors could give a short explanation.*

**Response:** Given the complexity of the experimental system, which requires the elaborate generation and maintenance of defined metacestode vesicles, screening procedures of more than ten compounds can either only focus on different concentrations for defined time points or different time points when only one concentration is employed. Since Echinococcus stem cells divide within 3-4 days (PMID:24602211) and since the appearance of fully differentiated cells (which relates to cellular turnover in metacestode vesicles) requires even longer, we routinely prefer the second approach. In typical hit-to-lead assays, it is common practice to use 10 micromolar as a starting concentration and to determine IC50 values only after having obtained promising hits. This is what we have done. Hence, to keep the response short, we overall prefer longer incubation times over testing different concentrations for a first screening. We now modified the manuscript and included in Fig. 1 not only the results after 3 d incubation but also after 7 d incubation. We modified the legend accordingly.

*Lane 552: The results on metacestode integrity are clearly demonstrated. However, it is not clear to the reader what type of mature vesicles were assessed. How long have these vesicles been in culture, are they derived from stem cell cultures, did they already have protoscoleces, and is there a difference between older and younger vesicles? I can see that the methodology was referenced, and I am sure the authors have this information.*

**Response:** We have now added more information to the M&M section concerning type, age, and fertility of vesicles. Just as brief information for the reviewer: for inhibitor assays we routinely use isolates that have lost the capacity of protoscolex production. This is because metacestode tissue in humans only rarely produces protoscoleces, because such vesicles can easier be monitored (and grow to larger size) than fertile vesicles, and because we intend to measure effects on metacestode tissue, and not on protoscoleces. Yes, there are certain differences between larger and smaller vesicles in inhibitor sensitivity since smaller vesicles are usually more sensitivity. The germinative layer of smaller vesicles is usually not as ‘thick’ and the larger volume of ‘hydatid fluid’ often quenches the inhibitor effects. For comparisons, we therefore utilize vesicles of comparable size and age (which results, of course, also in more elaborate handling of the cultures).

According to the guidelines for submission of this journal, ‘methods should be described in sufficient detail to repeat the experiments’. Furthermore, it says that ‘well-established methods are to be referenced’. In our opinion, the description of the methods in the revised version is adhering to this.

*Also, the assessment is based on visualization, which is good enough. When doing so, however, it is advised that this is done a blinded way (e.g. the one doing the assessment did not know what he/she is looking at). Any information on that should be given.*

**Response:** Of course, we inspect and analyze results of inhibitor assays not only by one, but by several persons. This is part of good laboratory practice and this is now mentioned in the revised version. It should be mentioned here that visual inspection of structural vesicle integrity is generally not a matter of ‘interpretation’. A damaged vesicle can easily be identified and we have clear criteria for counting such vesicles as damaged.

*Did the authors also consider to use another read-out found in the literature such as PGI or alkaline phosphatase assay?*

**Response:** Of course, we did consider this – and not only for this work but also for numerous other articles in the past. However, we never found that these tests outmatch visual inspection in smaller scale analyses. PGI or alkaline phosphatase assessment have clear advantages in medium- to high-throughput screens (i.e. hundreds to thousands of compounds), which was the reason why they have originally been developed, but they are indirect. Once these tests had been used for initial screen to narrow down sample size, visual inspection should be used to ascertain that vesicle damage indeed occurred. Since we focussed on smaller sample size, after employing a novel bioinformatic method for high-throughput screening (!), we solely relied on visual tests.

*Incubation for 28 days: I might have missed that, but were there medium changes and at what interval? Is it known how long these drugs are stable under culture conditions? Please keep in mind that it is very unlikely that drug levels remain stable over longer periods in vivo.*

**Response:** Maybe we did not explain it clearly enough and maybe it is not sufficient to cite previous work. If so, we apologize. At least we clearly stated that we change medium every three to four days. Of course, we then also add fresh inhibitor (otherwise it would not make sense). No, we do not know how stable each of the inhibitors is under these conditions. We assume that after addition of the inhibitors to the culture the concentration will be declining until the medium is changed next – and we think this assumption is reasonable. So, over time we assume that the average inhibitor concentration in the medium is below the concentration we originally (and during every medium change) added. We modified the text in M&M to clearly state when inhibitor was added. And we thank the reviewer for the advice to keep in mind that inhibitor concentrations can decline over time.

*Lane 590: clearly, these inhibitors exert detrimental effects on metacestodes. However, to what extent is this relevant?*

**Response:** It is relevant for target validation! It shows that Pim kinase inhibitors are active against the parasite and since the majority of amino acids critical for binding of these compounds to Pim kinases are also conserved in EmPim it indicates that EmPim inactivation probably has detrimental effects on Echinococcus. In other words, it indicates that EmPim is a target the inhibition of which can kill the parasite. We consider this highly relevant! By first showing that the parasite has a Pim kinase, by then showing that the critical amino acids for inhibitor binding are conserved, and by then showing that the inhibitors do have detrimental effects on the parasite, we thought the relevance is made clear. In this particular case we did not see any reason to modify the text of the original version, unless the other reviewers or the editors also state that they misunderstood our wording and/or our line of argumentation.

*It would be interesting to get a bit more information on the PK properties of these compounds. As one has undergone clinical trials at least with one of these compounds, there should be information on whether these concentrations showing an effect against E. multilocularis will ever be achieved in vivo. The concentration range that is active here appears rather high, and one then wonders whether the effects we see here are not based on off-target effects (even not necessarily based on inhibition of another kinase) that might occur. I am not questioning the results per se, but this aspect could be discussed.*
**Response:** First of all, we explicitly mentioned and discussed possible off-target effects of the inhibitors – and we even named possible off-targets. Second, we did not propose Z991902128 as a drug, but as a hit compound on the basis of which drugs could be developed. Third, even in this case we do not consider the active concentration range of the compounds in this study ‘rather high’. In previous in vitro studies using albendazole, the currently used drug against echinococcosis, which, according to Amman et al. (see argument of this reviewer above), can cure the disease in 1/3 of cases, in vitro concentrations of 30 micromolar (10 ug/ml ABZSO) had to be used (PMID:10223913, PMID:11451682). And this was albendazole-sulfoxide, the active compound, not pure albendazole. In the case of mebendazole, concentrations of 1-10 micromolar were active (PMID:9593125), which is still within the range used in the present study. In plasma of treated patients, albendazole sulfoxide levels between 3- and 10-fold lower are routinely measured (see e.g. PMID:30745388, PMID:21332573). So, given that we do not even suggest Z196138710 as a drug against echinococcosis and given that we find concentrations of 3 micromolar of our experimental compound active against the parasite’s stem cells, we cannot quite follow the reviewer’s critique that we used ‘rather high’ concentrations.

Anyhow, we searched the literature intensely for in vivo data on SGI-1776 and found several references, which we included in the revised version. This work shows that much (about 95%) of the drug is bound to human plasma protein but that concentrations of 3 micromolar of free (unbound) compound are achieved in the plasma of mammals. This is the concentration range which was required to see in vitro effects. We incorporated this information into the manuscript and further discussed the potential of Z196138710 as an anti-AE agent (lines 777 – 801).

*Lane 602: is the cause for the interruption of the heart rhythm (long QT syndrome) based on a specific interaction of SGI-776 with the the human Ether-à-go-go-Related Gene (hERG)? This is a common stumbling step in drug development. Clearly, the in silico approach appears to be a novel and highly versatile tool to obtain novel molecules such as Z196138710 with profound activities and in this case more specificity, and the results obtain are encouraging in terms of efficacy and specificity, but I would advise the authors to look into hERG inhibition of their novel compound early on.*

**Response:** Yes, it was. We discuss this in the revised version and in the future we will surely consider this.

*Lane 656: Screening of mammalian cells employed HepG2 and HEK293T cells, which are immortalized cell lines. It would be advisable to also assess cultures of non-immortalized cells or primary cell cultures of different origin.*

**Response:** Why is this advisable? Pim kinases are regulators of the cell cycle, are overexpressed in tumor cell lines, and these cell lines heavily depend on the activity of PIM kinases (referenced in our work). In particular, HEK293 and HepG2 cell lines were shown to be very sensitive to these inhibitors (also referenced in our work). Hence, if we want to measure effects on PIM kinases, these immortalized cell lines would be the primary choice. This is the reason why we chose them. Of course, there is always another cell line which could be tested, or another primary cell system, or alternative culture conditions and so on. But what exactly is the qualified reason to suggest non-immortalized cells in the context of this work? Why should these ‘alternative’ cell systems perform better in what we want to measure – i.e. effects of inhibitors on Pim kinases – than cellular systems in which the dependence on PIM kinases has already been demonstrated? Since the reviewer was not very specific concerning the nature of non-immortalized cells which could outmatch the systems we used, we would be grateful for advice.

*Reviewer #3: This is an interesting manuscript in which the authors identified the proto-oncogene EmPIM kinase as a promising target for anti-AE chemotherapy. The apparently well performed in situ hybridization assays indicated its expression in parasite stem cells. By yeast two-hybrid assays, the authors showed interaction of EmPIM with E. multilocularis CDC25, suggesting an involvement of EmPIM in parasite cell cycle regulation.
To improve compound specificity for EmPIM, the authors applied a high throughput in silico modelling approach, leading to the identification of compound Z196138710. Which, when applied to in vitro cultured metacestode vesicles and parasite cell cultures, proved equally detrimental as SGI-1776 and CX-6258, but displayed significantly reduced toxicity towards human HEK293T and HepG2 cells.
Overall, the work is interesting and relevant as a potential alternative for future anti-AE chemotherapy.

However, I have some doubts that in my opinion, merit some consideration/reflection.*

*Major:
To the light of the results presented here, the compound Z196138710 seems to be promissory in echinococcosis therapy.
However, I wonder if a more complete curve (with more than 3 points) could be performed for this compound in terms of viability, intact vesicles or regenerated vesicles. I´m not suggesting to do 3 curves, but only one which could allow to get an IC50 value or a clearer picture about the nature of the inhibition.*

**Response:** This is a valid objection by the reviewer. Indeed, IC50 values would be helpful. We therefore carried out additional experimentation as outlined in more detail below. We could not do so, however, in all aspects mentioned by the reviewer since at present compound Z196138710 is simply not available. The production facilities of Enamine Ltd., which synthesize Z196138710, are located in Kyiv, Ukraine, which currently is war zone. The last delivery batch of Z196138710 we obtained dated from January 2022 and was largely used up for the final experiments leading to this manuscript. The last update from Enamine Ltd dates from May 11^th^ (<https://enamine.net/news-events/1354-enamine-operation-update-may-11>) and states that production of compounds is about to be resumed, but so far it is unclear when Z196138710 will be available again. We asked several times (including intensely last month) but could not get definitive information.

Using up the last batch of Z196138710, we performed for the revised version IC50 studies on human cell lines (SGI-1776 and CX-6258 compared to Z991902128). These studies confirmed that the IC50 of Z991902128 against human cells is more than 10-fold higher than those of the known inhibitors (please see S4 Figure and text of revised version). As concerning IC50 tests on metacestode vesicles and primary cells, which would require significant higher amounts of inhibitor, we will have to wait until Z196138710 is available again. Although definite IC50 values are thus missing, we still sticked to our interpretation that Z991902128 is equally detrimental to Echinococcus larvae as SGI-1776 and CX-6258, and we hope the reviewer can accept this.

*I wonder if any information exists about the plasma levels of kinase inhibitors in mammals. If the answer to the previous question is affirmative and based on the dose response curve obtained for Z196138710, Could the plasma levels of kinase inhibitors be in the range necessary for therapeutic intervention of Echinococcosis?*   

**Response:** We already addressed this point in our response to the critique of reviewer #2 (see above). We did not take up respective information for a broad panel of kinase inhibitors, but concentrated on SGI-1776, which is subject to the topic of this work. Hence, according to previous work, such plasma levels can be achieved. We added respective information to the manuscript and discussed it accordingly (see text lines 777 - 801).

*Have the authors attempted to perform silencing of PIM in PC to evaluate the relevance of this kinase in cell viability and MV formation?*

**Response:** Yes, we made on attempt. We did not see phenotypes, but we achieved transcript knockdown to 85% only, which is surely not enough to deprive Echinococcus cells from EmPim. It should be noted that RNAi in Echinococcus is still in its infancy and that a stable phenotype has, to our knowledge, only be observed in one case (knocking down a microRNA gene; PMID:33750964). From our own unpublished work we know that RNAi can lead to significant transcript knockdown and to phenotypes. This, however, so far only in the case of unstable gene products (secreted factors and proteins which have rapid turnover). This is not expected in the case of EmPim. Therefore, we did not try a second attempt (with other siRNAs). Due to the very limited knockdown, we did not take up these results in the manuscript and we hope the reviewer can accept this.

--------------------
